# Use of an Additional Updated 2023–2024 COVID-19 Vaccine Dose for Adults Aged ≥65 Years: Recommendations of the Advisory Committee on Immunization Practices — United States, 2024

**DOI:** 10.15585/mmwr.mm7316a4

**Published:** 2024-04-25

**Authors:** Lakshmi Panagiotakopoulos, Monica Godfrey, Danielle L. Moulia, Ruth Link-Gelles, Christopher A. Taylor, Kevin Chatham-Stephens, Oliver Brooks, Matthew F. Daley, Katherine E. Fleming-Dutra, Megan Wallace

**Affiliations:** ^1^National Center for Immunization and Respiratory Diseases, CDC; ^2^Watts Healthcare Corporation, Los Angeles, California; ^3^Institute for Health Research, Kaiser Permanente Colorado, Denver, Colorado.

**Summary**

**What is already known about this topic?**

In September 2023, the Advisory Committee on Immunization Practices (ACIP) recommended updated (2023–2024 Formula) COVID-19 vaccination for all persons aged ≥6 months.

**What is added by this report?**

On February 28, 2024, ACIP recommended that all persons aged ≥65 years receive 1 additional dose of any updated (2023–2024 Formula) COVID-19 vaccine (i.e., Moderna, Novavax, or Pfizer-BioNTech).

**What are the implications for public health practice?**

Adults aged ≥65 years should receive an additional dose of the updated (2023–2024 Formula) COVID-19 vaccine to enhance their immunity and decrease the risk for severe COVID-19–associated illness.

## Abstract

COVID-19 remains an important public health threat, despite overall decreases in COVID-19–related severe disease since the start of the COVID-19 pandemic. COVID-19–associated hospitalization rates remain higher among adults aged ≥65 years relative to rates in younger adults, adolescents, and children; during October 2023–January 2024, 67% of all COVID-19–associated hospitalizations were among persons aged ≥65 years. On September 12, 2023, CDC’s Advisory Committee on Immunization Practices (ACIP) recommended updated (2023–2024 Formula) COVID-19 vaccination with a monovalent XBB.1.5-derived vaccine for all persons aged ≥6 months to protect against severe COVID-19–associated illness and death. Because SARS-CoV-2 continues to circulate throughout the year, and because of the increased risk for COVID-19–related severe illness in persons aged ≥65 years, the protection afforded by updated vaccines against JN.1 and other currently circulating variants, and the expected waning of vaccine-conferred protection against disease, on February 28, 2024, ACIP recommended all persons aged ≥65 years receive 1 additional dose of the updated (2023–2024 Formula) COVID-19 vaccine. Implementation of these recommendations is expected to enhance immunity that might have waned and decrease the risk for severe COVID-19–associated outcomes, including death, among persons aged ≥65 years.

## Introduction

Since June 2020, CDC’s Advisory Committee on Immunization Practices (ACIP) has convened 39 public meetings to review data and consider recommendations related to the use of COVID-19 vaccines (*1*). On September 12, 2023, ACIP recommended that all persons aged ≥6 months receive updated (2023–2024 Formula) monovalent, XBB.1.5 component (updated) COVID-19 vaccination to protect against severe COVID-19–associated illness and death (*2*).

As of February 3, 2024, approximately 6.7 million COVID-19–associated hospitalizations and 1.1 million COVID-19–associated deaths had occurred in the United States (*3*). Although the overall risk for COVID-19–associated hospitalization and death has decreased, severe illness related to COVID-19 continues to be a public health problem, especially among older adults. COVID-19–associated hospitalization rates remain higher among adults aged ≥65 years relative to rates among younger adults, adolescents, and children. During October 2023–January 2024, 67% of all COVID-19–associated hospitalizations were among persons aged ≥65 years (*4*). Further, COVID-19 death rates during January 1, 2023–January 31, 2024, were highest among adults aged ≥75 years, followed by adults aged 65–74 years (*5*,*6*). Whereas approximately 98%–99% of the U.S. population has measurable antibody titers against SARS-CoV-2 from infection, vaccination, or both (hybrid immunity), adults aged ≥65 years are less likely to have immunity resulting from infection (including immunity from infection only or hybrid immunity), compared with adults aged 30–49 years and 50–64 years (*7*). In addition, immunosenescence, the age-related decline in the functioning of the immune system, results in a less complete immune response to novel antigens and a reduced ability to develop robust immunity after infections or vaccination (*8*). The pool of naive T-cells diminishes with age, and this insufficient naive T-cell pool affects the ability to generate neutralizing antibody responses and cytotoxic T-cells in response to SARS-CoV-2 (*9*). 

Thus, adults aged ≥65 years are more likely than are younger adults, adolescents, and children to rely upon vaccination to increase immunity that might have waned and might need more frequent vaccine doses to maintain protection. Coverage with the updated COVID-19 vaccine among adults aged ≥65 years was 42% as of February 3, 2024 (*10*,*11*). Adults in this age group are more concerned about COVID-19 disease and had higher confidence in COVID-19 vaccine safety and vaccine importance than did younger adults (*5*). A nationally representative survey conducted during November 2023–January 2024 indicated that 68.4% of adults aged ≥65 years who had received an updated COVID-19 vaccine dose definitely would get another updated vaccine if it were recommended, 27.2% probably would or are unsure if they would get another updated vaccine, and 4.4% said they probably or definitely would not. COVID-19 vaccines are currently on the commercial market, but access-related barriers and disparities in vaccine coverage remain (*5*); in the absence of any recommendations for an additional dose, access to vaccine would be limited among persons unable to pay out of pocket for the vaccine.*

On February 28, 2024, ACIP voted to recommend that all persons aged ≥65 years receive 1 additional dose of any updated COVID-19 vaccine (i.e., Moderna, Novavax, or Pfizer-BioNTech). This recommendation was based on continuing SARS-CoV-2 circulation throughout the year, increased risk for severe illness attributable to COVID-19 in adults aged ≥65 years, protection provided by the updated vaccines against JN.1 and other currently circulating variants, the expected waning of SARS-CoV-2 immunity, and additional implementation considerations, including facilitating clear communication and equitable access to vaccine (*5*).

## Methods

In 2018, ACIP adopted the Evidence to Recommendations framework to guide the development of vaccine recommendations. Since November 2023, the ACIP COVID-19 work group met seven times to discuss the current policy question, i.e., whether adults aged ≥65 years should receive an additional dose of updated COVID-19 vaccine. Work group membership included ACIP voting members, representatives of ACIP ex officio and liaison organizations, and scientific consultants with expertise in public health, immunology, medical specialties, and immunization safety and effectiveness. Work group discussion topics included COVID-19 disease surveillance and epidemiology; COVID-19 vaccination coverage; and the safety, effectiveness, feasibility of implementation, and cost effectiveness of COVID-19 vaccines. This report summarizes the ACIP recommendation for an additional dose of the updated COVID-19 vaccine for persons aged ≥65 years and the rationale, including evidence reviewed by the work group and presented to ACIP (https://www.cdc.gov/vaccines/acip/recs/grade/covid-19-additional-dose-adults-etr.html).

## Vaccine Effectiveness and Safety

No clinical trial immunogenicity data on an additional dose of the updated COVID-19 vaccines exist; however, the initial dose elicits a robust neutralizing antibody response and provides protection against JN.1 and other circulating variants (*12*,*13*). Early vaccine effectiveness (VE) estimates demonstrate that updated COVID-19 vaccination provided increased protection against symptomatic SARS-CoV-2 infection and COVID-19–associated emergency department and urgent care visits and hospitalization, compared with receipt of no updated vaccine dose (*12**,**14*). Although these early VE estimates show no substantial waning, based on data on effectiveness of original and bivalent COVID-19 vaccines, waning of vaccine-conferred immunity is expected. Effectiveness of an additional dose in older adults has been demonstrated for previously recommended additional original COVID-19 vaccine doses (*15*). Among adults aged ≥50 years who were eligible to receive a second original monovalent mRNA COVID-19 vaccine booster dose, VE against COVID-19–associated emergency department and urgent care encounters during the SARS-CoV-2 Omicron BA.2/BA.2.12.1 period ≥120 days after the third dose was 32% but increased to 66% ≥7 days after the fourth dose. VE against COVID-19–associated hospitalization ≥120 days after the third dose was 55% but increased to 80% ≥7 days after the fourth dose (*15*). In addition, in a large cohort of nursing home residents during circulation of SARS-CoV-2 Omicron subvariants, receipt of a second original monovalent mRNA COVID-19 booster dose ≤60 days earlier was 74% effective against severe COVID-19–related outcomes (including hospitalization or death) and 90% effective against death, compared with receipt of a single booster dose (*16*). 

COVID-19 vaccines have a favorable safety profile as demonstrated by robust safety surveillance during 3 years of COVID-19 vaccine use (*17*). Anaphylactic reactions have rarely been reported after receipt of COVID-19 vaccines (*18*). A rare risk for myocarditis and pericarditis exists, predominately in males aged 12–39 years (*19*). No new safety concerns have been identified for the updated COVID-19 vaccine (*5*). Among adults aged ≥65 years, overall reactogenicity after COVID-19 vaccination is less frequent and less severe than among adolescents and younger adults (*20*). A statistical signal for ischemic stroke after Pfizer-BioNTech bivalent mRNA COVID-19 vaccine was detected in the CDC Vaccine Safety Datalink among persons aged ≥65 years, and information about this detection has been presented at previous ACIP meetings. Ongoing efforts to evaluate the signal have not identified any clear and consistent evidence of a safety concern for ischemic stroke with bivalent mRNA COVID-19 vaccines either when given alone or when given simultaneously with influenza vaccines (*21*). A recent VE study indicated that the bivalent COVID-19 vaccine was 47% effective in preventing COVID-19 related thromboembolic events (ischemic stroke, myocardial infarction, and deep vein thrombosis) among persons aged ≥65 years (*22*).

## Cost Effectiveness

ACIP considered whether an additional dose of updated COVID-19 vaccine in persons aged ≥65 years is a reasonable and efficient allocation of resources. The societal incremental cost-effectiveness ratio (ICER) for an additional dose of COVID-19 vaccine in persons aged ≥65 years was $255,122 per quality-adjusted life year saved for the base case estimate. ICER values were sensitive to probability of hospitalizations, costs, and seasonality assumptions. Estimates of ICER values that approximate cost effectiveness for those with higher risk for COVID-19–associated hospitalization, such as persons with underlying conditions or those aged ≥75 years, were more favorable (*23*).

## Recommendation for Use of an Additional Updated COVID-19 Vaccine Dose in Persons Aged ≥65 Years

On February 28, 2024, ACIP recommended that all persons aged ≥65 years receive 1 additional dose of any updated COVID-19 vaccine (i.e., Moderna, Novavax, or Pfizer-BioNTech).^†^ This additional dose should be administered ≥4 months after the previous dose of updated COVID-19 vaccine. For initial vaccination with Novavax COVID-19 vaccine, the 2-dose series should be completed before administration of the additional dose. Because Novavax COVID-19 vaccine is currently authorized under Emergency Use Authorization, the recommendation for the updated Novavax COVID-19 vaccine is an interim recommendation.

## Persons Aged ≥65 Years with Moderate or Severe Immunocompromise

Persons aged ≥65 years who are moderately or severely immunocompromised, have completed an initial series, and have received ≥1 updated COVID-19 vaccine dose should receive 1 additional updated COVID-19 vaccine dose ≥2 months after the last dose of updated vaccine. Further additional doses may be administered, guided by the clinical judgment of a health care provider and personal preference and circumstances. Any further additional doses should be administered ≥2 months after the last COVID-19 vaccine dose. Additional clinical considerations, including detailed schedules and tables by age for all age groups and vaccination history for those who are or are not moderately or severely immunocompromised, are available at https://www.cdc.gov/vaccines/covid-19/clinical-considerations/covid-19-vaccines-us.html.

## Reporting Vaccine Adverse Events

Adverse events after vaccination should be reported to the Vaccine Adverse Event Reporting System (VAERS). For licensed COVID-19 vaccines administered to persons aged ≥12 years, reporting is encouraged for any clinically significant adverse event even when whether the vaccine caused the event is uncertain, as well as for vaccination errors. For COVID-19 vaccines given under Emergency Use Authorization, vaccination providers are required to report certain adverse events to VAERS. Additional information is available at https://vaers.hhs.gov or by telephone at 1-800-822-7967.

## Footnotes

* Section 2713(a)(2) of the Public Health Service Act, as added by section 1001 of the Affordable Care Act, implemented at 26 CFR 54.9815–2713(a)(1)(ii), 29 CFR 2590.715–2713(a)(1)(ii), and 45 CFR 147.130(a)(1)(ii). This requirement does not apply to grandfathered health plan coverage under section 1251 of the Affordable Care Act, implemented at 26 CFR 54.9815–1251, 29 CFR 2590.715–1272, and 45 CFR 147.140.

† ACIP voted (11 to one with one abstention) to recommend that persons aged ≥65 years should receive an additional dose of updated (2023–2024 Formula) COVID-19 vaccine.
